# Furosemide stress test and interstitial fibrosis in kidney biopsies in chronic kidney disease

**DOI:** 10.1186/s12882-020-01721-z

**Published:** 2020-03-06

**Authors:** Jesús Rivero, Francisco Rodríguez, Virgilia Soto, Etienne Macedo, Lakhmir S. Chawla, Ravindra L. Mehta, Sucheta Vaingankar, Pranav S. Garimella, Carlos Garza, Magdalena Madero

**Affiliations:** 1 Nephrology Department, National Institute of Lung Disease Ismael Cosio Villegas , Mexico City, Mexico; 2 Nephrology Department, National Institute Cardiology Ignacio Chávez, Juan Badiano No. 1, 14080-Tlalpan, Mexico City, Mexico; 3 Division of Nephrology, University of California, San Diego, CA USA

**Keywords:** Interstitial fibrosis, Uresis, Kidney biopsy, Furosemide stress test

## Abstract

**Background:**

Interstitial fibrosis (IF) on kidney biopsy is one of the most potent risk factors for kidney disease progression. The furosemide stress test (FST) is a validated tool that predicts the severity of acute kidney injury (especially at 2 h) in critically ill patients. Since furosemide is secreted through the kidney tubules, the response to FST represents the tubular secretory capacity. To our knowledge there is no data on the correlation between functional tubular capacity assessed by the FST with IF on kidney biopsies from patients with chronic kidney disease (CKD). The aim of this study was to determine the association between urine output (UO), Furosemide Excreted Mass (FEM) and IF on kidney biopsies after a FST.

**Methods:**

This study included 84 patients who underwent kidney biopsy for clinical indications and a FST. The percentage of fibrosis was determined by morphometry technique and reviewed by a nephropathologist. All patients underwent a FST prior to the biopsy. Urine volume and urinary sodium were measured in addition to urine concentrations of furosemide at different times (2, 4 and 6 h). We used an established equation to determine the FEM. Values were expressed as mean, standard deviation or percentage and Pearson Correlation.

**Results:**

The mean age of the participants was 38 years and 44% were male. The prevalence of diabetes mellitus, hypertension and diuretic use was significantly higher with more advanced degree of fibrosis. Nephrotic syndrome and acute kidney graft dysfunction were the most frequent indications for biopsy. eGFR was inversely related to the degree of fibrosis. Subjects with the highest degree of fibrosis (grade 3) showed a significant lower UO at first hour of the FST when compared to lower degrees of fibrosis (*p* = 0.015). Likewise, the total UO and the FEM was progressively lower with higher degrees of fibrosis. An inversely linear correlation between FEM and the degree of fibrosis (r = − 0.245, *p* = 0.02) was observed.

**Conclusions:**

Our findings indicate that interstitial fibrosis correlates with total urine output and FEM. Further studies are needed to determine if UO and FST could be a non-invasive tool to evaluate interstitial fibrosis.

**Trial registration:**

ClinicalTrials.gov NCT02417883.

## Background

Chronic kidney disease (CKD), particularly in advanced stages, is considered one of the most serious public health problems worldwide [1, 2].For a long time, efforts have been made understand the risk factors associated to the development of kidney fibrosis, since it is considered one of the most potent risk factors for CKD progression, independent from the CKD etiology, as it represents a final common pathway from injury [3].The tubules and interstitial compartment together constitute approximately 80% of the renal mass and is, therefore, more than a complex support system structures [4–6].There are three main recognized histological structures that can be assessed for fibrosis: glomerulosclerosis, vascular sclerosis andtubular atrophy/tubulointerstitial fibrosis (IFTA) [7, 8].IFTA is the final result of an anatomic and functional imbalance where the kidney insults exceed the known compensatory mechanisms of repair where different mediators generate the transition from the normal tubular structure to myofibroblasts and elements of the extracellular matrix [9, 10]. At this point changes are irreversible and therefore strongly associated to kidney disease progression [11].Trials performed in patients undergoing kidney transplantation suggest that IFTA is strongly implicated with graft failure, progression, and increased mortality [12–14].

For several decades the evaluation of kidney function has been based primarily on measurement glomerular filtration rate (mGFR) or on equations based either on creatinine or cystatin C where glomerular filtration rate is estimated [15–29].These methods however do not measure tubular function nor estimate the degree of tubular atrophy or interstitial fibrosis. Some evaluation tools to assess tubular function have been tested such as dilution capacity, concentration [18–31] and urinary acidification [31–33], in addition to excreted fraction of sodium and urea (FENA and FEUN) [34–36], however these have not been widely used in clinical practice. The furosemide stress test (FST) has demonstrated to predict the severity and acute kidney injury (especially at 2 h) in critically ill patients and has proved to be superior to urinary biomarkers [37].Furosemide must be actively transported by the human organic anion transporters (hOAT) in the proximal tubule into the tubular lumen in order to be excreted. Since the urinary excretion of furosemide is mediated by the proximal tubule organic anion transport system, we evaluated if the urinary excretion of furosemide and the associated diuretic response were related to the severity of tubulointerstitial damage determined in renal biopsies. We set out to assess the timing and magnitude of furosemide excretion and urine output and determine if these measures may be informative with regard to IF in patients undergoing kidney biopsy.

## Methods

### Methods

**Fig. 1:**
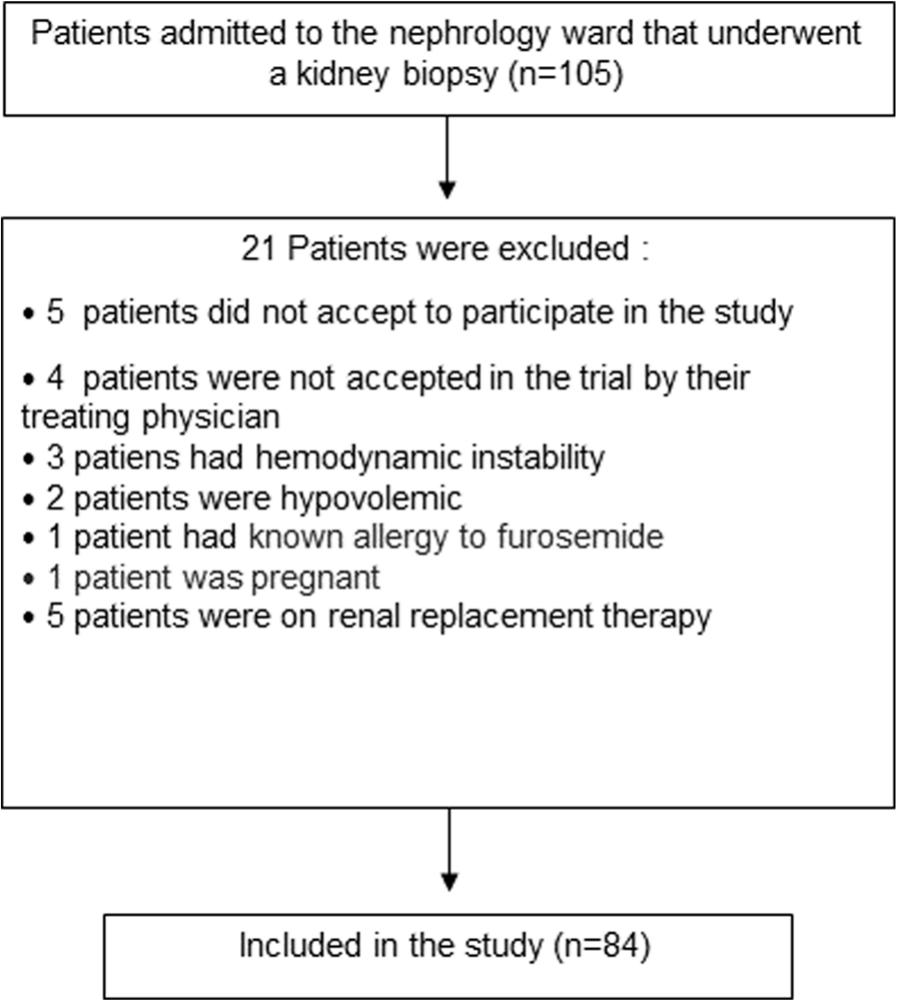
Patients Flow Diagram

We included inpatients that were admitted to the hospital for a kidney biopsy with a clinical indication (allograft and native). Patients were admitted to the nephrology ward at the Instituto Nacional de Cardiologia Ignacio Chavez. Patients needed to be older than 18 years, had a creatinine eGFR ≥15 ml/min by CKD-EPI [38] and had to be hemodynamically stable. Subjects were excluded if they did not accept to participate in the study or if the medical team did not feel the patient was suitable for the study, if they had a known allergy to furosemide, were on renal replacement therapy or were pregnant (Fig. 1). The study was conducted between April 2015 and August 2017.

The research was approved by the institutional research ethics committee and registered with the number 15–920. Written informed consent was obtained from all participants.

## Intervention

Furosemide test was performed during the hospitalization for the kidney biopsy. Most of the FST were done 1 day after the kidney biopsy was performed in full agreement by the medical team. In order to avoid urine measurement errors in those patients who did not have a bladder catheter on the day of the test, a post-voiding bladder volume measurement was performed using pelvic sonography.

All the 84 kidney biopsies were done at the bedside of the patient using ultrasound guidance (Sonosite®). For the kidney biopsies we used a Biopsy Gun (BARD Magnum®) and needles, procedures were done under local anesthesia.

### Furosemide stress test

**Fig. 2:**
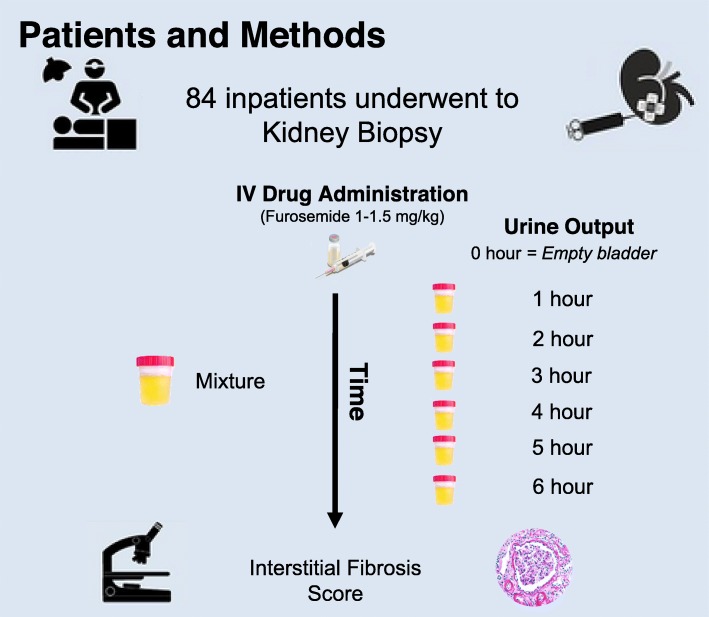
Furosemide Stress Test Procedure

Intravenous furosemide was administered at a dose of 1 mg /kg if subjects had not been exposed to diuretics during the previous 7 days and at a dose of 1.5 mg/kg if they have been exposed to loop diuretics within the 7 days prior to the study [37]. Medical surveillance and strict hourly UO quantification was done. UO was collected at baseline and every hour until the sixth hour (Fig. 2). Volume status was evaluated by the treating physician, patients that were hypovolemic or had hemodynamic instability were excluded from the study (*n* = 5), 76 subjects were euvolemic and 8 subjects were hypervolemic. Fluid replacement on euvolemic subjects as given on a 1:1 cc based on the previous hourly urine output. Hypervolemic patients did not receive intravenous fluid replacement.

### Histopathological analysis

#### Kidney specimen

The kidney biopsy was processed by the pathology department at the Instituto Nacional de Cardiología Ignacio Chávez. All kidney biopsy tissues were considered sufficient for histopathologic evaluation. An experienced renal pathologist blinded to the FST results described the degree of interstitial fibrosis, through direct visualization of light microscopy, with emphasis on the staining of Masson’s Trichrome. In addition, fibrosis percentage was assessed by anoptic lector machine (Morphometry) using the Analyzer Olympus BX51 Microscope, image Software Image-PN- Plus 6 and Camera: VF Evolution C (half Cybernetics). This technique has been accepted as a standard as it is more reproducible [39]. IF score was categorized into three groups according to fibrosis percentage: Grade I < 25%, Grade 2, 26–50% and grade 3 > 50%. Since tubular atrophy (TA) and IF are so highly correlated, TA is not routinely described in our pathology reports.

*Urine Furosemide Excretion:* UO was collected at every hour on the hour for 6 h and stored separately in aliquots. Furosemide analyses were determined in individual samples collected at hour 2 (0–2 h), 4 (2–4 h), 6 (4–6 h) and in a mix of all urines.

### HPLC analysis of furosemide in urine samples

Urine samples were purified using Oasis MCX Cartridge (Part#186000254 Oasis MCX 3 cc cartridge 60 mg 30 μm). The cartridge was washed with 2 ml methanol followed by 2 ml of milliQ water and loaded with the urine sample (0.5 ml adjusted to pH 6.0 with 2 M HCl and diluted with 0.5 ml of MilliQ water). Next, the cartridge was washed with 1 ml of 2% formic acid to remove acidic compounds followed by 1 ml of methanol to remove neutrals and the bound furosemide was eluted with 1 ml of 5% ammonium hydroxide in methanol. The eluted ammonium hydroxide fraction was dried in a speed-vac, reconstituted in 100ul of MilliQ water and quantified by validated HPLC test [40]. The HPLC column used was Thermo Scientific Acclaim 120 T, 3X150mm, C18, 3um, 120A, with TSK 120 T guard cartridge. The flow rate was 0.5 ml/min, column temperature 40 °C and run time 15 min. The isocratic elution solvent was 30% acetonitrile and 70% potassium phosphate monobasic buffer (10 mM, pH 3.85). Sample volume 50ul was injected and furosemide was detected at UV 233 nm, retention time 6.3 min.

We estimated the furosemide excreted mass equation (FEM) expressed as percentage using the following equation:

$$ \mathrm{FEM}\%=\frac{UrineFurosemide\kern0.5em x\  Urinary\ Volume}{Administred\kern0.5em Furosemidedose}\times 100 $$

### Statistical analysis

All values were expressed as means, standard deviation, and percentages. Results were expressed as average, standard deviation (SD) or as proportions as appropriate. The comparisons were made using chi [2] for proportions and by means of student T test for independent samples (comparison between groups) according to the response to furosemide. The Pearson test was used to determine the correlation between degree of fibrosis and response to FST. We used statistical program SPSS 16 version.

## Results

The mean age was 38 years, 44% were male and the mean eGFR was 64 ± 42 ml/min/1.72m^2^. The presence of diabetes mellitus, hypertension and use of diuretics was predominantly higher in grade III fibrosis. No episodes of hypotension or hipokalemia occurred during the study. As expected, eGFR decreased as interstitial fibrosis increased (Table 1). Nephrotic syndrome and acute kidney graft dysfunction were the most frequent indications for biopsy.

**Table 1:** Baseline Characteristics

Variable	Kidney allograft (*n* = 30)	Native kidneys (*n* = 54)	Total group (*n* = 84)	*P*
Age (y)	34.6 ± 14.8	40.9 ± 15	38.6 ± 15.1	0.071
Gender (M/F) N (%)	18 (60) / 12 (40)	19 (35.2) / 35 (64.8)	37 (44) / 47 (56)	0.028*
Baseline eGFR (ml/min/1.73m^2^)	47.2 ± 26.8	73.2 ± 45.6	63.9 ± 41.6	0.001*
Serum Albumin (g/dl)	3.8 ± 0.7	2.9 ± 0.8	3.2 ± 0.9	0.000*
Urinary protein excretion (g/g creatinine)	1.62 ± 2.63	4.55 ± 4.52	3.47 ± 4.16	0.002*
Diabetes Mellitus N (%)	23 (76.7)	–	23 (76.7)	0.000*
Hypertension N (%)	–	20 (37)	20 (37)	0.000*
Systemic lupus erythematosus N (%)	1 (3.3)	21 (38.9)	22 (26.2)	0.000*
Interstitial Fibrosis N (%)				
Grade I	13 (43.3)	32 (59.3)	45 (53.6)	0.003*
Grade II	16 (53.3)	11 (20.4)	27 (32.1)	0.003*
Grade III	1 (3.3)	11 (20.4)	12 (14.3)	0.003*
Combined	25.3 ± 15.4	28.7 ± 22.3	27.5 ± 20.1	0.409

Data are expressed as mean ± standard deviation, frequency or percentage as appropriate

*eGFR* estimated glomerular filtration rate, *AKI* acute kidney injury, *FEM* furosemide excreted mass, *IFI* interstitial fibrosis < 25%, *IFII* interstitial fibrosis 26–50%, *IFIII* interstitial fibrosis > 50%

*p < 0.05 kidney allograft vs. native kidneys

We observed that subjects with grade 3 fibrosis showed a significant lower urine volume at hours 1, 4 and total urine when compared with grades I and II (155 mL ± 181 vs 316 mL ± 262 vs 328 ± 353) (Table 2).

**Table 2:** Urine Output and Furosemide Excreted Mass

Variable	Combined *n* = 84	IF Grade I *n* = 45	IF Grade II *n* = 27	IF Grade IIIn = 12	*P*
Urine measurements					
**Uresis 1 h**	313 ± 296.7	316.4 ± 261.9	328.5 ± 352.7	155 ± 181	0.015
Uresis 2-h (mL)	355 ± 254	387 ± 284	374 ± 216	260 ± 229	0.413
Uresis 4-h (mL)	250 ± 212	291 ± 202	241 ± 243	125 ± 106	0.054
Uresis 6-h (mL)	200 ± 179	195 ± 179	228 ± 182	155 ± 180	0.492
Total Uresis (mL)	1509 ± 779	1599 ± 790	1591 ± 816	995 ± 415	0.045
FEM-2 h (%)	5.5 ± 6.5	6.8 ± 7.4	5.1 ± 5,7	1.6 ± 1.7	0.049
FEM-4 h (%)	2.9 ± 4.0	4.0 ± 4.6	1.9 ± 2.9	0.8 ± 1.3	0.012

**Fig. 3:**
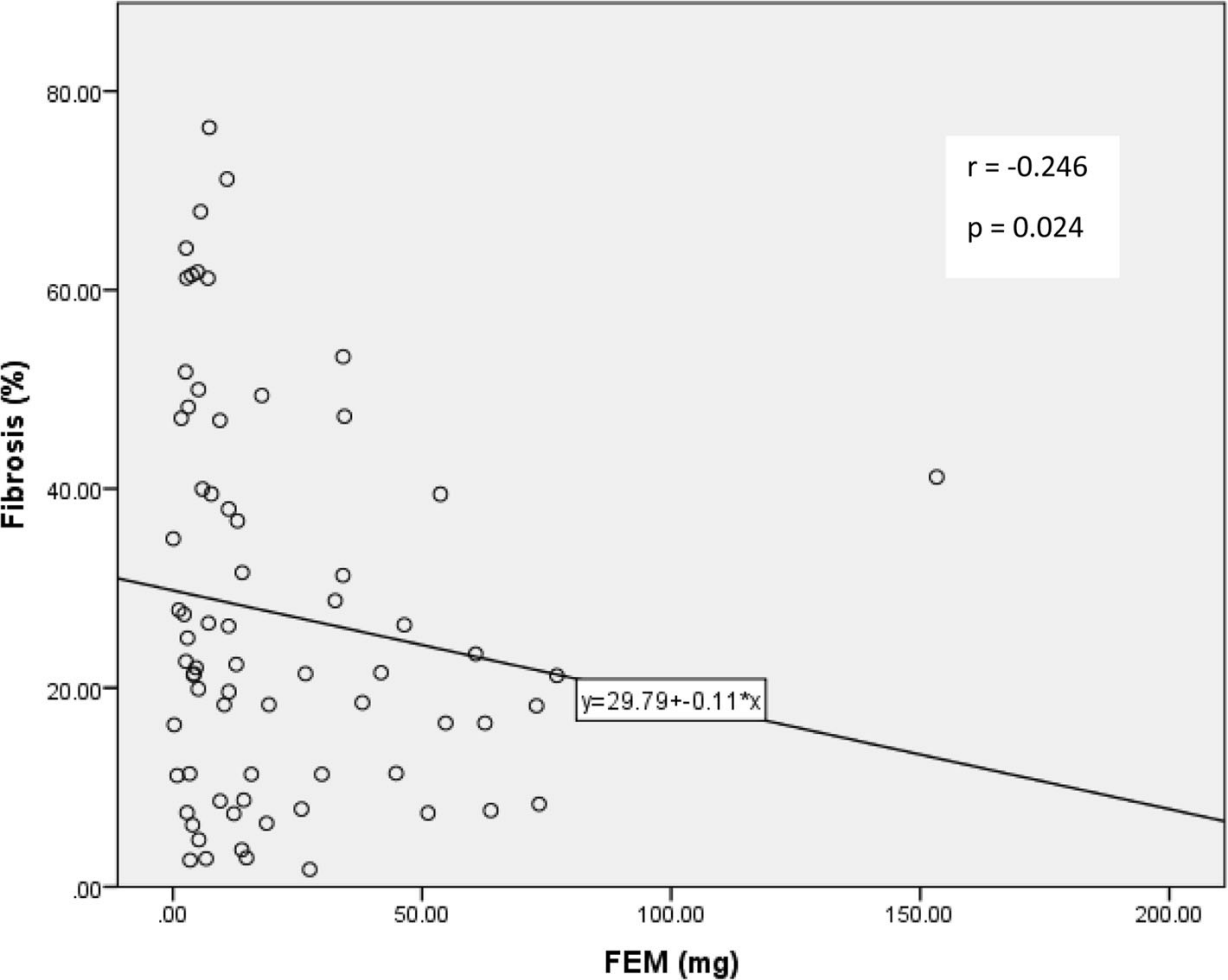
Correlation analysis of interstitial fibrosis and FEM

The (FEM) was progressively lower with higher degrees of fibrosis, at hours 2 and 4 (1.6 and 0.8 for grade III fibrosis vs 6.8 and 4.0) (Table 2 and Fig. 3). When analyzed separately the correlation between FEM2 and fibrosis was significant (*p* = 0.04) in the native kidneys but not in the transplant group (*p* = 0.26) (Table 3).

**Table 3:** Pearson’s correlation coefficient between MFP and FEM2, eGFR and total uresis for native kidneys and allografts

MFP Total (*n* = 84)	FEM-2 (mg)	eGFR (mL/min/1.73 m2)	Total uresis (mL)
Natives (n = 54)	- 0.27 *(p = 0.043)*	*- 0.56 (p = 0.000)*	*- 0.34 (p = 0.012)*
Allografts (n = 30)	- 0.21 *(p = 0.264)*	- 0.34 *(p = 0.059)*	- 0.09 *(p = 0.613)*

*MFP* morphometric fibrosis percentage, *FEM2* furosemide excreted mass at 2 h, *eGFR* estimated glomerular filtration rate

We did not find a significant difference between the measurement of subjective fibrosis and morphometry (27.7 ± 19.8 *p* = 0.795 vs 26.2 ± 17.2 *p* = 0.608).

A sub analysis was performed to evaluate the behavior of the FEM at different times with respect to the cut-off point that showed the best area under the curve in previously reported cohorts in the literature with acute kidney injury (200 ml of uresis), identifying that the FEM at the second hour was the measurement that best correlated with this cut point. (Table 4).

**Table 4:** Mass of Excretion of Furosemide according to urine output cutoff at hour 2

Measurement time point	Positive (Urine Output > 200 ml)	Negative (Urine Output < 200 ml)	*P*
FEM-2	7.2 ± 6.9 (*n* = 61)	1.1 ± 2.2 (*n* = 23)	< 0.0001
FEM-4	2.9 ± 3.8 (n = 61)	2.7 ± 4.5 (n = 23)	0.847
FEM-6	1.8 ± 2.8 (*n* = 54)	2.2 ± 3.7 (*n* = 18)	0.592
FEM Mixture	21.2 ± 22.0 (*n* = 51)	20.8 ± 34.9 (*n* = 20)	0.949

An inverse correlation between UO and the degree of fibrosis (the greater the response in urinary output, the lower the degree of fibrosis) was observed (quadratic correlation of 0.072, per each ml change in UO there was a 0.2 change in fibrosis).

### Sensitivity analysis

**Fig. 4:**
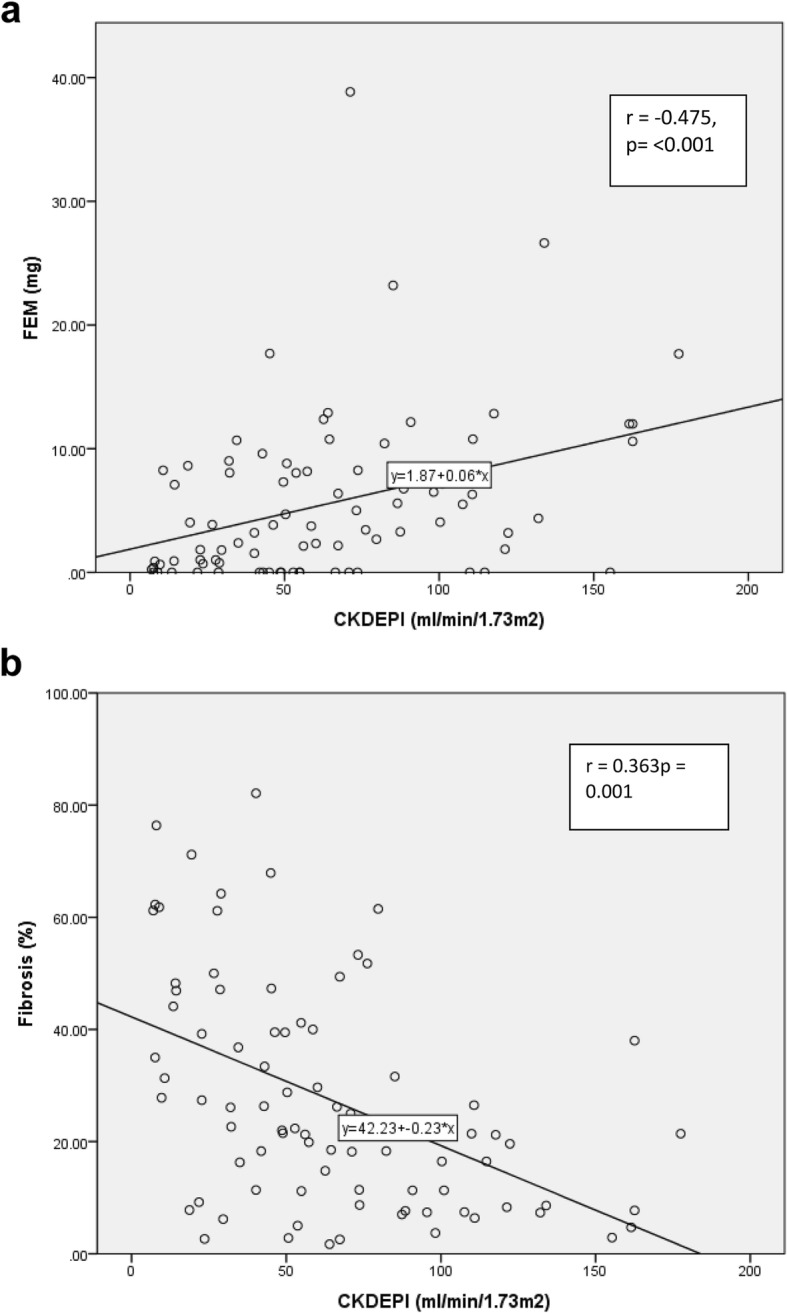
**a** and **b** FEM2, fibrosis score and baseline eGFR

Since albumin is necessary for the secretion of furosemide, we evaluated the Pearson correlation between serum albumin with urine output and FEM. There was a significant correlation between serum albumin and total UO (*p* = .008) (At higher serum albumin we observed higher UO) but there was no significant correlation between albumin and FEM (*p* = 0.64). Likewise, there was no correlation between levels of proteinuria and FEM (*p* = 0.48). When analyzed separately, the correlation between proteinuria and FEM in native versus transplant kidneys was not different (*p* = 0.25 vs *p* = 0.86, respectively). As expected, there was a significant correlation between eGFR (CKDEPI) and FEM_2_ (*p* = 0.001) and between eGFR and fibrosis (p = < 0.001) (Fig. 4a, b). Finally, since most of the allograft biopsies were performed for allograft dysfunction we evaluated the rate of acute kidney injury in our population. Creatinines were assessed 3 months before and 3 months after the study using a non-parametric test (Wilcoxon range test) for comparisons. In all the comparisons of related samples we found *p* values greater than 0.1 (p = NS). Results were no different between allograft and native kidneys.

## Discussion

In our study we were able to establish the association between a functional tubular assessment with the FST and the degree of interstitial fibrosis obtained in kidney biopsies. Urine volume correlated with the degree of fibrosis and the excreted mass of furosemide decreased as fibrosis increased. To our knowledge this is the first study to establish a tubular anatomic-functional relationship.

We found that the furosemide excreted mass at 2 h (FEM_2_) correlated with low urine output during the same time frame. The FEM_2_ is conceived to function similarly to the forced exhaled volume (FEV_1_) in pulmonary function tests. A healthy person can expire most of the air from their lung in 1 s. Similarly, a healthy person with a full complement of functioning nephrons can rapidly eliminate furosemide into the urine. However, when tubular dysfunction is present as the case with tubular-interstitial fibrosis, the fibrosed kidney loses its capacity to efficiently move furosemide out of the plasma and into the tubular lumen. This type of functional testing may form the basis of assessing tubular function in a non-invasive fashion.

The evaluation of the tubular functional capacity however is rarely done in the clinical setting and the estimation of kidney function for patients with CKD is primarily based on the eGFR. There are different ways to evaluate the tubular function, such as the kidney concentration capacity, acidification capacity, calculation of relative clearance of a substance such as beta 2 microglobulin [41] or creatinine. In addition, urine uromodulin which is the most abundant protein secreted by the tubules has been described as a marker of tubular function and has been associated with eGFR decline and cardiovascular outcomes [42].

Some of the first studies to evaluate tubular function were done by our group and others 30 years ago. In those studies, the tubular functional capacity was evaluated looking at the difference between creatinine clearance and inulin clearance [43]. In those studies, the glomerular reserve was assessed with a protein challenge. These studies were conducted in three groups of patients: healthy patients, kidney donors and patients with CKD demonstrating in the latter a lower ability to increase GFR before protein administration and a limited creatinine tubular clearance [44]. However, these tests remain a research tool and can be cumbersome due to the protein loading as such have limited clinical applicability.

The idea to evaluate tubular function with the use of intravenous furosemide relies on the ability of the drug to be secreted in the proximal tubule. In order for furosemide to increase urine output, furosemide must be actively secreted into the proximal lumen, and the functions of the thick ascending limb, luminal patency and collecting duct should be preserved. The furosemide stress test has been used in clinical practice only in the context of acute kidney injury and has been shown to predict outcomes in this population. A standard dose of intravenous furosemide was delivered to critically ill patients with KDIGO stage I or II AKI, and then urine output response was assessed. A 2-h urine output < 100 ml/h in response to a furosemide challenge predicts progression to KDIGO stage III AKI within 14 days with a receiver operator characteristic area under of the curve (AUC) of 0.87 [37]. This test performed better than many of the urinary biomarkers used to predict AKI. Subsequent studies of FST in AKI have demonstrated that the FST performance is robust and performs consistently for the assessment of AKI progression and recovery [45–48].However, none these studies measured the excreted mass of furosemide.

The assessment and monitoring of CKD are primarily focused on GFR and degree of proteinuria. While not demonstrated in this study, the FST may offer a non-invasive functional tool for the clinician to assess tubular function in patients with kidney disease. Nonetheless, the FST appears to offer novel information in these patients.

Our study has several strengths. First, the study explores a novel concept of tubular function and its association with interstitial fibrosis in kidney biopsies. Second, the amount of fibrosis on the kidney biopsies was assessed also by morphometry removing the potential observation bias by the pathologist. Third, in addition to urine output, we measured the urine excreted mass of furosemide. Lastly, this analysis does demonstrate that the FST offers more information as compared to classic GFR assessment.

Our study also has limitations. First this is a cross sectional study and we do not have data on the impact of the FST over kidney function decline over time. Second, the strength of the association among the different variables analyzed (interstitial fibrosis, FEM, CKDEPI and total urine output) was significant only in the subgroup of native kidneys compared to the group of renal transplantation, probably in relation to a greater renal mass and the larger sample size of the group of native kidneys Finally, although statistically significant, the correlations were not strong*.*

## Conclusions

In conclusion, our findings support that interstitial fibrosis correlates with FST with both total urine output and the furosemide excreted mass. This could be established as non-invasive tool to evaluate interstitial fibrosis and may offer more prognostic information over eGFR and proteinuria alone. Further longitudinal studies are needed to establish if FST is associated with kidney function decline over time.

## Data Availability

The datasets used and/or analysed during the current study are available from the corresponding author on reasonable request.

## Abbreviations

CKD: Chronic kidney disease
FEM2: Furosemide Excreted mass at 2 h
FST: Furosemide stress test
GFR: Glomerular filtration rate
IF: Interstitial fibrosis
MFP: Morphometric fibrosis percentage
UO: Urine output
